# Medium- and High-Entropy Rare Earth Hexaborides with Enhanced Solar Energy Absorption and Infrared Emissivity

**DOI:** 10.3390/ma17081789

**Published:** 2024-04-12

**Authors:** Hongye Wang, Yanyu Pan, Jincheng Zhang, Kaixian Wang, Liyan Xue, Minzhong Huang, Yazhu Li, Fan Yang, Heng Chen

**Affiliations:** 1 College of Chemistry and Materials Science, Fujian Normal University, Fuzhou 350117, China; xmwanghongye@fjirsm.ac.cn; 2 Fujian Institute of Research on the Structure of Matter, Chinese Academy of Sciences, Fuzhou 350002, China; xmpanyanyu@fjirsm.ac.cn (Y.P.); xmzhangjincheng@fjirsm.ac.cn (J.Z.); xmwangkaixian@fjirsm.ac.cn (K.W.); xmxueliyan@fjirsm.ac.cn (L.X.); xmmzhuang2022@fjirsm.ac.cn (M.H.); xmliyazhu@fjirsm.ac.cn (Y.L.); 3 Xiamen Institute of Rare Earth Materials, Haixi Institute, Chinese Academy of Sciences, Xiamen 361021, China; 4 Fujian Science & Technology Innovation Laboratory for Optoelectronic Information of China, Fuzhou 350108, China; 5 Xiamen Key Laboratory of Rare Earth Photoelectric Functional Materials, Xiamen Institute of Rare Earth Materials, Haixi Institute, Chinese Academy of Sciences, Xiamen 361021, China; 6 Key Laboratory of Rare Earths, Ganjiang Innovation Academy, Chinese Academy of Sciences, Ganzhou 341000, China

**Keywords:** concentrated solar power (CSP), solid particle, medium- and high-entropy ceramics (ME/HECs), rare earth hexaborides, high solar absorptivity, high infrared emissivity

## Abstract

The development of a new generation of solid particle solar receivers (SPSRs) with high solar absorptivity (0.28–2.5 μm) and high infrared emissivity (1–22 μm) is crucial and has attracted much attention for the attainment of the goals of “peak carbon” and “carbon neutrality”. To achieve the modulation of infrared emission and solar absorptivity, two types of medium- and high-entropy rare-earth hexaboride (ME/HEREB_6_) ceramics, (La_0.25_Sm_0.25_Ce_0.25_Eu_0.25_)B_6_ (MEREB_6_) and (La_0.2_Sm_0.2_Ce_0.2_Eu_0.2_Ba_0.2_)B_6_ (HEREB_6_), with severe lattice distortions were synthesized using a high-temperature solid-phase method. Compared to single-phase lanthanum hexaboride (LaB_6_), HEREB_6_ ceramics show an increase in solar absorptivity from 54.06% to 87.75% in the range of 0.28–2.5 μm and an increase in infrared emissivity from 76.19% to 89.96% in the 1–22 μm wavelength range. On the one hand, decreasing the free electron concentration and the plasma frequency reduces the reflection and ultimately increases the solar absorptivity. On the other hand, the lattice distortion induces changes in the B–B bond length, leading to significant changes in the Raman scattering spectrum, which affects the damping constant and ultimately increases the infrared emissivity. In conclusion, the multicomponent design can effectively improve the solar energy absorption and heat transfer capacity of ME/HEREB_6_, thus providing a new avenue for the development of solid particles.

## 1. Introduction

The efficient utilization of solar energy has become a key means of achieving the goals of “carbon neutrality” and “carbon peak” [1]. Concentrated solar power (CSP) is a type of electricity generation that captures solar energy and uses it directly as a heat source for the electricity cycle. For third-generation CSP, molten salts, solid particles, and gases are candidates for the capture of solar energy [2,3]. As a key component of a solid particle solar receiver (SPSR) [4,5], solid particles not only heat themselves from concentrated sunlight but also act as a heat transfer medium to transfer heat to the back-end for power generation. Direct solar absorption based on solid particles and their use as heat transfer mediums (HTMs) has attracted increased interest as potential high-temperature CSP. The ideal particles should have high solar absorptivity (0.28–2.5 μm) and high infrared emissivity (1–22 μm) [6,7,8,9]. The absorption of solar radiation is an important part of the photothermal conversion process. High absorption in the visible and near-infrared spectra is essential for ensuring the efficient operation of solar concentrators. Moreover, high emissivity in the infrared range is necessary to exchange the absorbed energy of the falling particles with the fluid in the form of infrared radiation in the thermal exchanger. This approach simplifies the complexity of concentrated solar power systems and achieves the integration of heat absorption and thermal radiation in the particulate medium. Currently, various solid particles, including mullite [10], sand [11], quartz [9], silica sand [6,12,13], alumina [6,14,15], and olivine [16], can be used as heat transfer mediums (HTMs) in receivers [17]. However, current conventional particulate heat absorbers suffer from low heat absorption and heat transfer performance. Therefore, new materials with high solar absorptivity and high infrared emissivity need to be developed to solve the problems of heat absorption and transfer.

Rare-earth hexaborides (REB_6_) have a special crystal structure of the CsCl-type. On the one hand, the special boron network structure and the dominance of strong B–B bonds provide excellent properties of a low work function, high thermal stability, high mechanical strength, and high melting point [18,19,20,21]. On the other hand, the thermal, electrical, magnetic, and optical properties are significantly influenced by the presence of La metal atoms embedded within a stable boron octahedron network [22]. Elisa Sani et al. investigated the spectral reflectance and thermal emissivity of single-phase LaB_6_ and LaB_6_-ZrB_2_-SiC composites and found that LaB_6_ had the potential to be used as a high-temperature direct solar absorber [23]. LaB_6_ particles possess excellent optical properties and high thermal stability; thus, they are a suitable choice for SPSRs. However, the lower emissivity of LaB_6_ [23] does not meet the requirements of heat transfer mediums (HTMs). In order to simultaneously possess solar absorption and back-end heat dissipation, it is necessary for the material to have a high solar absorptance and infrared emittance. Therefore, the solar absorptance and infrared emittance of LaB_6_ need to be increased by other means.

In recent years, high-entropy ceramics (HECs), which are novel materials with no less than five equimolar metal cations or anions [24], have been the focus of much research. Various HECs have been successively designed and developed [21,25,26,27,28,29,30,31,32,33]. The use of highentropy provides a new strategy for tailoring materials properties. This approach is an effective means of modulating the emissivity of materials. Zhou et al. synthesized (La_0.2_Nd_0.2_Gd_0.2_Sm_0.2_Pr_0.2_)MgAl_11_O_9_ and found that it had a much higher infrared emissivity than LaMgAl_11_O_9_ [34]. Wang et al. synthesized La(Fe_0.2_Co_0.2_Ni_0.2_Cr_0.2_Mn_0.2_)O_3_ ceramics, which exhibited an emissivity of up to 92% in the range from 0.76 to 2.50 μm [35]. Song et al. designed and synthesized high-entropy transition metal disilicides and the higher infrared emissivity was due to the increase in phonon scattering caused by lattice distortion; this reduced the thermal and electrical conductivity of the lattice and ultimately increased its infrared emissivity [36]. Consequently, to control infrared emissivity and solar absorptivity, the high-entropy strategy is an effective method. However, the application of HEREB_6_ ceramic particles for solar energy absorption has not been reported. Therefore, the high-entropy design is expected to improve the solar absorptivity and infrared emissivity of LaB_6_, providing a new strategy for the development of SPSRs.

In this study, we aimed to improve the solar absorptivity and infrared emissivity by designing and synthesizing two ME/HEREB_6_ of (La_0.25_Sm_0.25_Ce_0.25_Eu_0.25_)B_6_ (MEREB_6_) and (La_0.2_Sm_0.2_Ce_0.2_Eu_0.2_Ba_0.2_)B_6_ (HEREB_6_) by a high-temperature solid phase method using a multicomponent design strategy. The phase composition and morphology of these HEREB_6_ were analyzed and the effects of composition and lattice distortion on their solar absorptivities and infrared emissivities were systematically investigated. Their solar absorptivity and infrared emissivity were systematically compared with those of single-phase LaB_6_. High entropy was found to be an effective means of overcoming the shortcomings of LaB_6_. The results show that ME/HEREB_6_ exhibited high infrared emissivity and high solar absorptivity; thus, a multicomponent design provides a novel approach to the development of solar energy materials.

## 2. Experimental

### 2.1. Composition Design

To demonstrate the influence of the multicomponent design on the solar absorption and infrared emissivity of metal hexaborides, using LaB_6_ as the base, MEREB_6_ and HEREB_6_ were designed and synthesized via a high-temperature solid-phase method based on the atomic size difference as the primary criterion. The choice of Ce is due to the small difference between the atomic radius and La, while the selection of Sm, Eu, and Ba is made to reduce the free electron concentration by introducing variable-valence ions or divalent ions.

The average size difference between medium- and high-entropy rare earth hexaborides can be expressed as Equation (1)

(1) $$\delta_{r}=\sqrt{\sum_{i=1}^{n}r_{i}{\left[1-r_{i}/(\sum_{i=1}^{n}x_{i}r_{i})\right]}^{2}}$$

where *n* is the number of metal components, *x_i_* is the molar fraction of the *i*th component of the metal, and *r_i_* represents the atomic radius of the *i*th component of the metal.

The configuration entropy of medium- and high-entropy rare earth hexaborides can be expressed as Equation (2)

(2) $$\Delta S_{conf}=-R\sum_{i=1}^{n}x_{i}\ln x_{i}$$

where R is the gas constant, *n* is the number of metal components, and *x_i_* is the molar fraction of the *i*th component of the metal.

The average size difference and configuration entropy are shown in Table 1, the average size differences between MEREB_6_ and HEREB_6_ are 2.58% and 3.95%, respectively. Moreover, the entropy values of MEREB_6_ and HEREB_6_ are 1.3863R and 1.6094R, respectively.

### 2.2. Materials and Methods

Three ceramic materials, LaB_6_, (La_0.25_Sm_0.25_Ce_0.25_Eu_0.25_)B_6_ (MEREB_6_), and (La_0.2_Sm_0.2_Ce_0.2_Eu_0.2_Ba_0.2_)B_6_ (HEREB_6_), were synthesized by a high-temperature solid-state reaction method. Commercially available lanthanum oxide (La_2_O_3_, ≥99.99%), samarium oxide (Sm_2_O_3_, ≥99.99%), cerium oxide (CeO_2_, ≥99.5%), europium oxide, (Eu_2_O_3_, ≥99.99%), barium carbonate (BaCO_3_, ≥99.95%), and boron carbide (B_4_C, ≥98%, ≥200 mesh) were purchased from Aladdin (Aladdin Biochemical Technology Co., Ltd., Shanghai, China) as raw materials. ME/HEREB_6_ powder was prepared by mixing four rare earth oxide powders and barium carbonate powder with B_4_C powder in the desired stoichiometric ratio. The initial stage of the boron carbide reduction process leads to the formation of rare earth borates as an intermediate phase with the reaction equation shown in Equations (3)–(8) [37], where RE denotes La, Sm, Ce, and Eu.

(3) $${4\mathrm{RE}}_{2}O_{3}{+7B}_{4}{C=4\mathrm{REBO}}_{3}{+\mathrm{REB}}_{6}+7C$$

(4) $${4\mathrm{REBO}}_{3}{+7C+5B}_{4}{C=4\mathrm{REB}}_{6}+12\mathrm{CO}↑$$

(5) $${(3)+(4):4\mathrm{RE}}_{2}O_{3}{+12B}_{4}{C=5\mathrm{REB}}_{6}+12\mathrm{CO}↑$$

(6) $${6\mathrm{CeO}}_{2}{+4B}_{4}{C=4\mathrm{CeBO}}_{3}{+2\mathrm{CeB}}_{6}+4C$$

(7) $${4\mathrm{CeBO}}_{3}{+4C+5B}_{4}{C=4\mathrm{CeB}}_{6}+9\mathrm{CO}↑+\frac{3}{2}O_{2}↑$$

(8) $${(6)+(7):6\mathrm{CeO}}_{2}{+9B}_{4}{C=6\mathrm{CeB}}_{6}+9\mathrm{CO}↑+\frac{3}{2}O_{2}↑$$

However, Ba, as a neighboring element of La, was prepared in this study using the same mechanism as REB_6_. Since BaO reacts readily with water and carbon dioxide in the air to form BaCO_3_, BaCO_3_ was chosen as the Ba source for the experiment. Therefore, the initial stage of its reaction may also form an alkaline earth borate [38] intermediate phase and the reaction equation is shown in Equations (9)–(11):

(9) $${2\mathrm{BaCO}}_{3}{+2B}_{4}{C=\mathrm{BaB}}_{2}O_{4}{+\mathrm{BaB}}_{6}+2C+2\mathrm{CO}↑$$

(10) $${\mathrm{BaB}}_{2}O_{4}{+2C+B}_{4}{C=\mathrm{BaB}}_{6}+3\mathrm{CO}↑+\frac{1}{2}O_{2}↑$$

(11) $${(9)+(10):2\mathrm{BaCO}}_{3}{+3B}_{4}{C=2\mathrm{BaB}}_{6}+5\mathrm{CO}↑+\frac{1}{2}O_{2}↑$$

In particular, the molar ratio of reactants is listed in Table 2.

These powders were weighed and transferred to an agate mortar in a stoichiometric ratio according to the composition of the target. Then, the mixture was ground by hand for 1 h until it was thoroughly mixed. The mixture obtained was kept at a pressure of 5 MPa for 30 s and then pressed into green bodies 20 mm in diameter. The green bodies were then placed in a graphite crucible in a tube furnace and argon gas was passed through the crucible for a period to ensure air removal. The green bodies were then heated to 1600 °C in an argon atmosphere at a heating rate of 5 °C/min and held for 2 h. The samples were cooled to 1000 °C (again at a rate of 5 °C/min) and then cooled in the furnace. Finally, all calcined samples were ground once again to obtain a fine powder.

### 2.3. Analysis and Characterization

The phase structures of LaB_6_ and ME/HEREB_6_ were identified with an X-ray diffractometer (XRD, Miniflex 600, Rigaku, Japan) over the range of 15–105° (2*θ* degree) with Cu Kα radiation (λ = 1.5406 Å) at a scanning rate of 1°/min and a step size of 0.02. Rietveld refinement was used to extract the phase structure information from the XRD data with the aid of Fullprof software 2024 [39]. The microstructure and elemental distribution of the samples were analyzed using field emission transmission electron microscopy (FTTEM, Talos F200s, Thermo Fisher Scientific, Waltham, MA, USA) in combination with an energy dispersive spectroscopy (EDS) system. The Raman spectra of the samples were collected with a LabRAM Aramis (Horiba Jobin Yvon S.A.S., Palaiseau, France) automated scanning confocal Raman microscope excited using a laser wavelength of 532 nm. The optical reflectance properties were measured with a UV–vis–NIR spectrophotometer (Carry 5000, Agilent Technologies Ltd., Santa Clara, CA, USA) equipped with a 110 mm InGaAs integrating sphere in the wavelength range from 0.28 to 2.5 μm; barium sulfate was used as a reference. The normal infrared emissivity of all of the samples in the range of 1–22 μm was measured using a dual-band infrared emissivity meter (IR-2, Chengbo Optoelectronics Technology Co., Ltd., Shanghai, China).

## 3. Results and Discussion

### 3.1. Phase Composition and Microstructure

**Figure 1:**
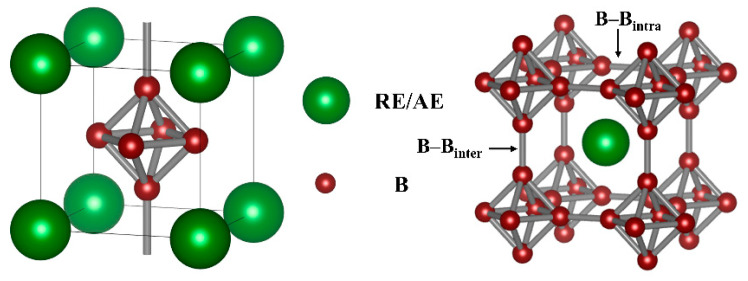
Crystal structure of REB_6_.

As shown in Figure 1, the crystal structure of CsCl-type rare earth hexaborides (REB_6_) is cubic with a space group of Pm-3 m (No. 221), the Wyckoff sites of rare earth in 1a (0, 0, 0), and boron elements in 6f (0.5, 0.5, z); here, the position parameter z of the B atom is related to the lattice parameter (a) and the B-B bond lengths [40]. Furthermore, the structure of REB_6_ is a compound consisting of ionic and covalent bonds, where the RE-RE bond is ionic, the RE-B bond is intermediate between ionic and covalent bonds, and the B-B bond is covalent [41]. Moreover, the strength of the σ-bonds between the octahedra (B-B_inter_) exceeds that of the τ-bonds within octahedra (B-B_intra_) [42]. In addition, the lattice parameters can be modified by varying the length of the inter-octahedral σ bonds (B-B_inter_) and intra-octahedral τ bonds (B-B_intra_) [43]. Changes in the B-B bond length can have a significant effect on the optical properties, resulting in an improvement in the infrared emissivity. The variations in the B-B_inter_ and B-B_intra_ distance spacings significantly impact on the optical properties, resulting in enhanced infrared emissivity. The optical properties of high-entropy REB_6_ are excellent and this improvement may be attributed to changes in the lattice distortion [36,44] and the free electron concentration. However, the boron sublattice in hexaborides is charge deficient and the number of deficient electrons has been experimentally and theoretically determined to be 2 [45]. Therefore, a metal cation with at least a +2 charge must be present to maintain electronic stability. However, the REB_6_ compound family includes trivalent rare earth ions such as La, Ce, Pr, and Nd, mixed-valent ions such as Sm (approximately 2.7), divalent ions such as Eu, and divalent alkaline earth elements such as Ca, Sr, and Ba [46,47,48]. Therefore, the free electron concentration can be reduced by introducing divalent or variable valence ions.

**Figure 2:**
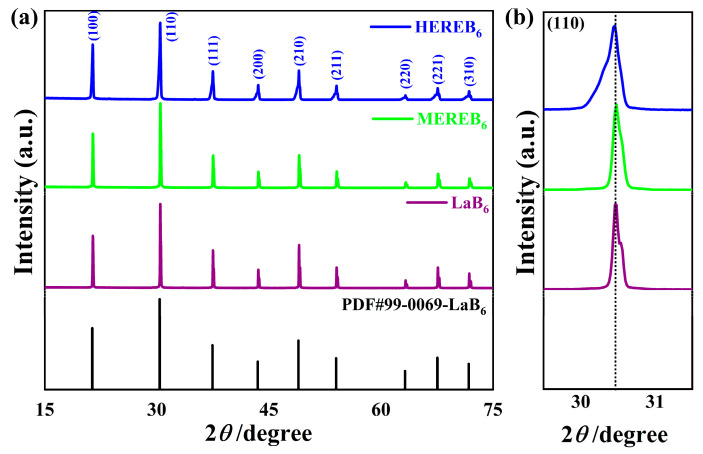
(**a**) XRD pattern of ME/HEREB_6_ and (**b**) partial enlargement of the (110) plane.

Figure 2a shows the XRD patterns of the synthesized LaB_6_, MEREB_6_, and HEREB_6_ powders together with the Standard ICDD/JCPDS cards of LaB_6_. No significant difference was observed between the XRD patterns of ME/HEREB_6_ and LaB_6_. The X-ray diffraction pattern of ME/HEREB_6_ shows a single solid solution phase. Figure 2b shows the partial enlargement of the (110) peak at 2*θ* values of 28.5–31.5°. Compared to the peak position of LaB_6_, the peaks of MEREB_6_ shifted toward higher 2*θ* values and HEREB_6_ shifted toward lower 2*θ* values. Thus, the lattice constant decreases or increases and these results are consistent with the data in Table 2, which show a reduction in cell parameters after refinement. Variations in the lattice parameters can be attributed to different group element designs, resulting in different degrees of lattice distortion. In addition, the broadening of the main peak of the XRD pattern is also observed, which may be due to grain refinement caused by elemental doping.

**Figure 3:**
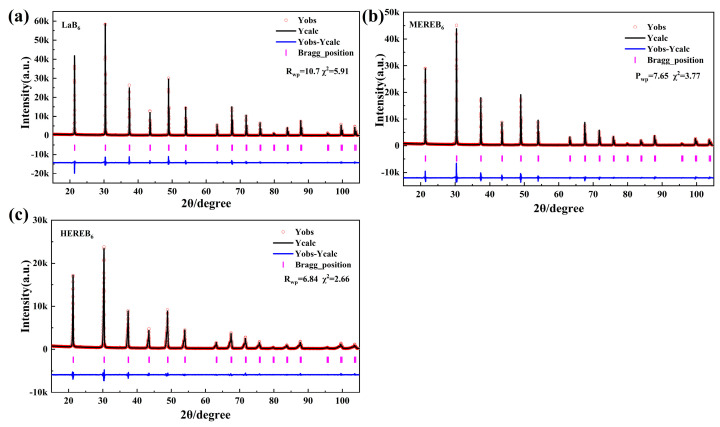
(**a**–**c**) Rietveld refinement of the XRD patterns.

The Rietveld method of crystal structure refinement [39] uses a whole-pattern profile fitting technique, which allows the crystal structure information to be obtained from powder X-ray diffraction data. Figure 3a–c displays the XRD Rietveld refinement results for LaB_6_, MEREB_6_, and HEREB_6_. Evidently, the calculated XRD patterns are in good agreement with the experimental patterns. The difference curves (Yobs-Ycal) in Figure 3b,c are smoothed and the χ^2^ values are less than 4%, indicating that the refinement results are reliable [49]. The difference curves (Yobs-Ycal) in Figure 3a are smoothed and have a slightly higher χ^2^ value (5.91%) because of the very high precision of the data collected [50]. Therefore, the results from this refinement are also reliable. Moreover, the refined unit-cell parameters and bond lengths for LaB_6_, MEREB_6_, and HEREB_6_ are listed in Table 3.

**Figure 4:**
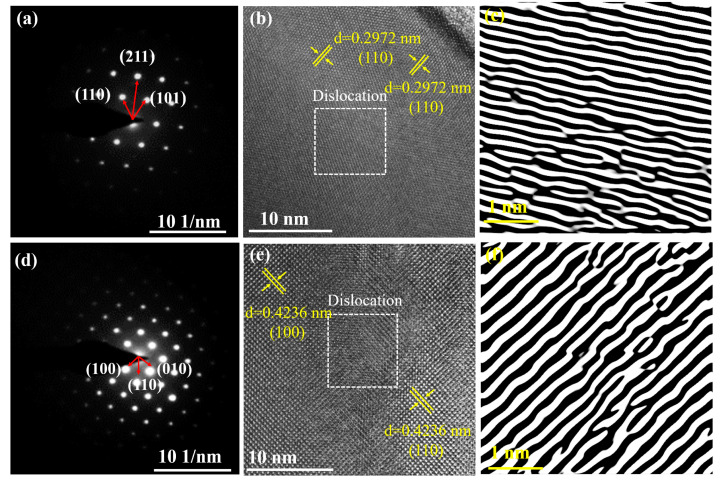
Samples of (**a**–**c**) MEREB_6_ and (**d**–**f**) HEREB_6_, with their electron diffraction patterns and corresponding inverse fast Fourier transform (IFFT) patterns.

**Figure 5:**
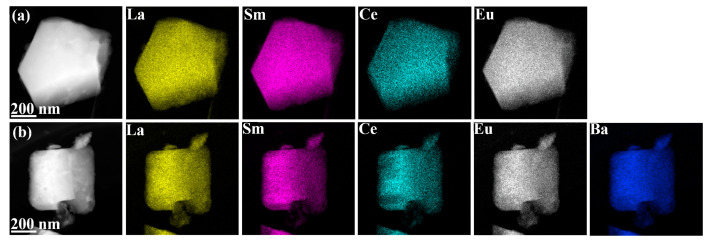
Compositional maps from EDS of (**a**) MEREB_6_ and (**b**) HEREB_6_.

Figure 4a,d shows the selected region electron diffraction (SAED) plots; here, the clear and regular diffraction spots confirm that MEREB_6_ and HEREB_6_ have a CsCl-type crystal structure and high crystallinity. Figure 4b,e shows the typical (110) and (100) lattice plane of the MEREB_6_ and HEREB_6_ powders with D-spacings of 0.2972 nm and 0.4236 nm, respectively. These values are similar to the values of 0.2938 nm (110) and 0.4158 nm (100) calculated from the XRD images. Figure 4c, f shows the inverse fast Fourier transforms (IFFTs) of the MEREB_6_ and HEREB_6_ images from the dislocations in the white boxed area in Figure 4b,e, respectively. Furthermore, linear dislocation lines, which exist alone or intertwined to form dislocation networks (as shown in Figure 4c,f), are observed [51,52]. The dislocations potentially occur due to the lattice distortion caused by the multi-component design. The MEREB_6_ and HEREB_6_ powders were further characterized by HR-TEM. Figure 5a,b shows the high-angle annular dark-field (HAADF) images and corresponding elemental distribution maps of MEREB_6_ and HEREB_6_. No apparent segregation of any of the elements was observed and this result provides further evidence of the formation of a uniform single-phase solid solution.

### 3.2. Lattice Distortion Analysis

**Figure 6:**
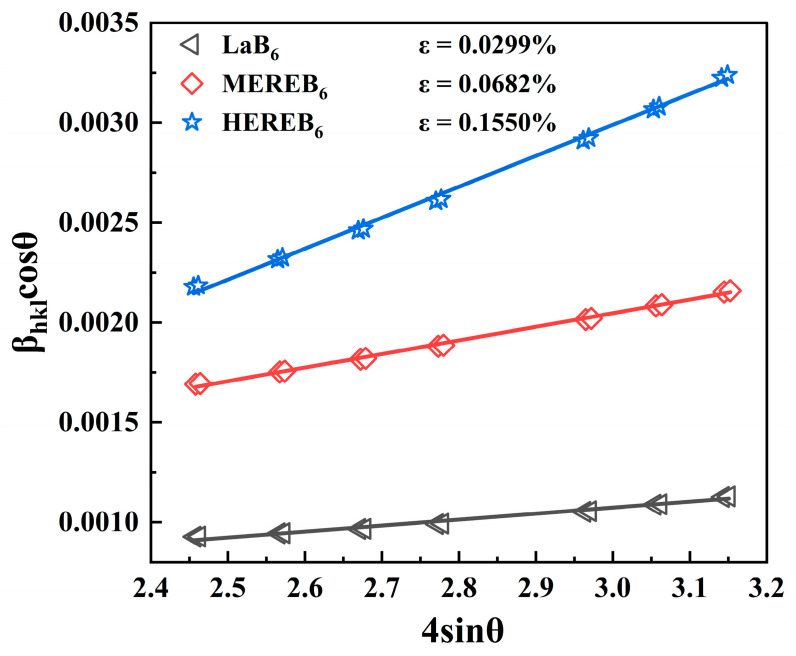
Lattice strains calculated based on Williamson–Hall analysis.

In theory, lattice distortion refers to the extent to which the positions of the constituent atoms deviate from the ideal lattice punctures [53]. In addition, fluctuations in the nature of chemical bonding in the crystal structure can also be considered part of the lattice distortion, such as the magnitude of changes in bond angles and bond lengths [54]. The variation in the B-B bond lengths is shown in Table 1. It has been reported that there is a relationship between the infrared emission properties of a material and its lattice strain; thus, to quantify the extent of the lattice distortion [44], the lattice strain of the samples was calculated using the Williamson–Hall formula (Equation (12)) [55]

(12) $$\beta \cos\theta =\frac{k\lambda }{D}+4\varepsilon \sin\theta$$

where *β* is the half-peak height and width, *θ* is the diffraction angle, *D* is the grain size, *λ* is the wavelength of the Cu Kα radiation source (λ = 1.5406 Å), and *ε* is the lattice strain. A straight line of *β*cos*θ* versus 4sin*θ* for the samples was fitted from the XRD data. The lattice strains (*ε*) of the samples were determined from the positive slopes. Figure 6 shows the calculated lattice strains based on Williamson–Hall analysis. A gradual increase in the lattice strain for LaB_6_, MEREB_6_, and HEREB_6_ was observed with values of 0.0299%, 0.0682%, and 0.1550%, respectively, as shown in Figure 6. Clearly, the lattice strain trend is in agreement with the lattice constants listed in Table 1.

**Figure 7:**
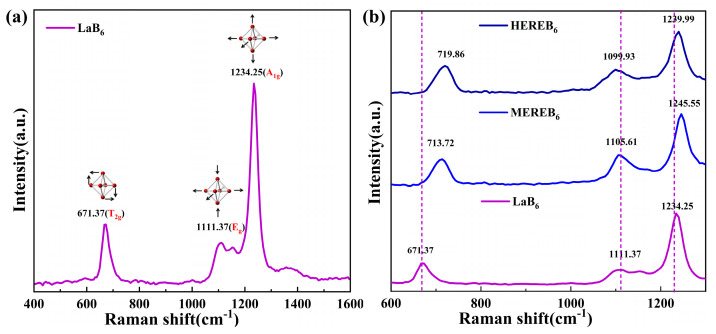
(**a**) Raman spectra of LaB_6_ and (**b**) Raman spectra of LaB_6_, MEREB_6_, and HEREB_6_.

Lattice distortion of the major factors influencing the properties of high-entropy materials. In addition, Raman spectroscopy can also verify lattice distortion [56]. The hexaborides of the rare earth elements have a cubic CsCl-type structure with a Pm-3 m space group. According to group theory, a set of allowed vibrational modes of the crystals in the Pm-3m structure is given by Equation (13) [57]:

(13) $$\Gamma =A_{1g}+E_{g}+T_{1g}+T_{2g}+2T_{1u}+T_{2u}$$

where *A*_1*g*_, *E_g_*, and *T*_2*g*_ denote the Raman active modes. Figure 7a shows the Raman spectrum of LaB_6_, where three main peaks were observed at 671.37 cm^−1^(*T*_2*g*_), 1111.37 cm^−1^(*E_g_*), and 1234.25 cm^−1^(*A*_1*g*_); the results were in agreement with previous reports [58,59]. The eigenvectors of these three intramolecular vibrations of the B_6_ molecules are shown in the inset of Figure 7a. In addition, *E_g_* and *A*_1*g*_ correspond to B–B stretching modes and the T_2g_ mode is a B–B–B valence angle bending mode of the boron lattice [57,58,60]. Simultaneously, the *T*_2*g*_ peak is blueshifted, the *E_g_* peak is redshifted, and the *A*_1**g**_ peaks are initially blueshifted and then redshifted, as shown in Figure 7b. Compared to single-phase LaB_6_, high-entropy hexaboride powders show a broadening of the Raman peak shape due to the lattice distortion and internal stress.

### 3.3. Solar Energy Absorption

**Figure 8:**
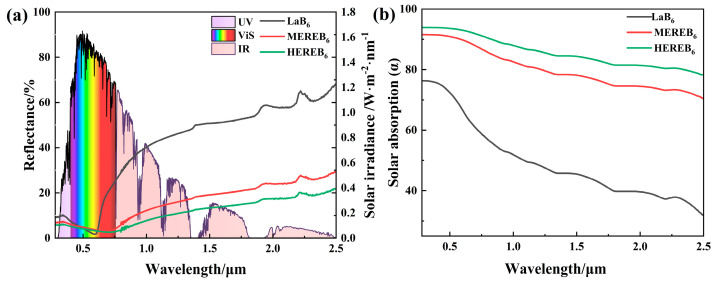
(**a**) Reflectance and solar irradiance spectra and (**b**) solar absorption rates of the LaB_6_, MEREB_6_, and HEREB_6_.

Figure 8a displays the reflectance and solar irradiance spectra of LaB_6_, MEREB_6_, and HEREB_6_. The reflectance spectra gradually decrease with the doping of rare earth and alkaline earth ions, indicating an increase in absorption. This is due to the change in the plasmon resonance with the free electron concentration. According to the literature, the plasma frequencies of LaB_6_, SmB_6_, CeB_6_, EuB_6_, and BaB_6_ are 4.94 eV, 2.61 eV, 4.90 eV, 1.32 eV, and 0.60 eV [61,62], respectively. With the exception of the divalent REB_6_ (EuB_6_ and YbB_6_), the divalent alkaline earth hexaborides (BaB_6_, SrB_6_, CaB_6_, etc.) and SmB_6_, the number of conduction band electrons per unit cell in trivalent rare-earth hexaborides is one and the Boron-2p bonding bands are filled with two electrons per mole [45]. The conduction band is formed by the rare-earth 5d-Boron 2p bonding band. On the other hand, SmB_6_ has only 0.6 to 0.7 electrons per mole due to a change in Sm valence, while divalent hexaborides have only a few conduction electrons [63]. Elemental doping reduces the free electron concentration and plasma frequency, resulting in a redshift of the absorbance band. This effectively regulates the near-infrared absorption of the material and enhances its solar energy absorption capacity. In addition, from the room temperature hemispherical reflectance R(λ), the total solar absorptance α can be calculated by Equation (14):

(14) $$\alpha =\frac{\int_{\lambda_{\min}}^{\lambda_{\max}}(1-R(\lambda ))\cdot S(\lambda )d\lambda }{\int_{\lambda_{\min}}^{\lambda_{\max}}S(\lambda )d\lambda }$$

where λ_min_ = 0.28 μm, λ_max_ = 2.5 μm, and S(λ) is the Sun emission spectrum, which is the standard solar spectral irradiance obtained from ISO Standard 9845-1(2022), AM1.5 [64]. The solar absorption rate calculated according to eq 1 is shown in Figure 8b. With the doping of rare earth and alkaline earth ions in the multicomponent design, the solar absorption rate of the samples gradually increased. The average solar absorption rate increased from 54.05% to 87.75%.

### 3.4. Infrared Emissivity

The infrared emissivity of a material is one of the most important indicators of its application value providing its ability for infrared radiation emission. According to Kirchhoff’s law [65], the following can be said:

(15) α+r+τ=1

where *α* is the absorptivity, *τ* is the transmittance, and *r* is the reflectance. Since the sample powders prepared for the test are dark purple and dark blue, they hardly transmit any light radiation, so their transmittance *τ* is zero, so Equation (16) can be simplified as follows:

(16) α+r=1

According to Kirchhoff’s law, the emissivity is equal to the absorbance when the material is in thermal Equation (17) as follows:

(17) α=ε

where *ε* is the infrared emissivity. It can therefore be deduced that for the infrared emissivity *ε*, the following is true:

(18) ε=1−r

**Figure 9:**
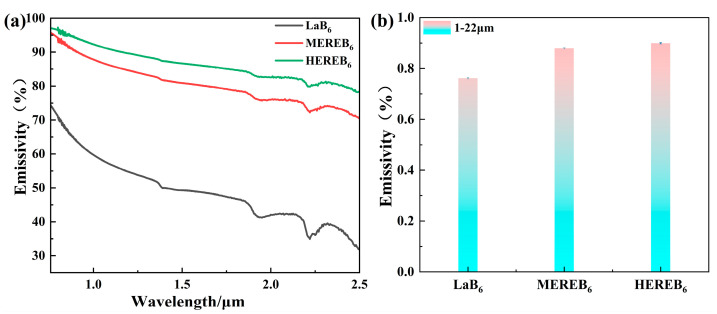
(**a**) Spectrum of the sample emissivity in the range of 0.76–2.5 μm and (**b**) the normal infrared emissivity in the range of 1–22 μm.

As shown in Figure 9a, HEREB_6_ exhibits an average infrared emissivity of 88.76% in the range of 0.76–2.5 μm. Notably, this improvement is remarkable and the emissivity is 1.65 times greater than that of LaB_6_ (53.64%). Moreover, the high-entropy design effectively addressed the low emissivity limitations of boride ceramics in this band. Figure 9b shows the normal infrared emissivity in the range of 1–22 μm. The infrared emittance of the sample surface at room temperature from 1 to 22 μm was measured by an active blackbody radiation source and the normal reflectance R of the sample surface was determined. According to Kirchhoff’s law, the absorptivity of the object is numerically equal to the emissivity, that is, σ = ε and the normal emissivity ε of the measured object in the infrared band was obtained. The infrared emissivity of the multicomponent design is significantly greater in the 1–22 μm band, with the highest emissivity for the HEREB_6_ samples reaching 89.96% in the range of 1–22 μm. Moreover, the emissivity of LaB_6_ is as high as 76.19%. These results indicate that LaB_6_ has an inherently high infrared emission capability. Clearly, the emissivity increases from 76.19% to 89.96% after the multicomponent design strategy with the rare earth and alkaline earth ions.

REB_6_ compounds are metal-like conducting ceramic materials with a high concentration of free electron gas containing bound and free electrons and their optical properties in the infrared region are determined by the in-band dielectric function of the dielectric function [36]. The intraband dielectric function is represented using Equation (19)

(19) $$\varepsilon_{\mathrm{int}ra}(\omega )=1-\frac{\omega_{D}^{2}}{\omega (\omega +i\eta )}$$

where *ω_D_* is the plasma frequency for intraband transitions (Equation (20)) and *η* is the damping constant of electrons.

(20) $$\omega_{D}^{2}=\frac{n_{e}e^{2}}{m_{eff}}$$

where *n_e_* is the concentration of the free carrier, *e* is the elementary charge, and *m_eff_* is the effective mass of the carrier.

In addition, the electrical conductivity is expressed as (Equation (21)) according to the Drude model for an electron gas.

(21) $$\sigma =\frac{ne^{2}\tau }{m_{e}}$$

where *n* denotes the electron density, *e* is the electron charge, *m_e_* is the electron mass, and *τ* is the relaxation time. The damping constant of the electrons, the reciprocal of the relaxation time *τ*, is determined by the electron scattering mechanism, which limits the electrical conductivity. In particular, the electrical conductivity exhibits a negative correlation with *η* [36,66]. However, much research has shown that high-entropy strategies reduce the conductivity of a material. Moreover, lattice distortion induces changes in the B–B bond length, leading to significant changes in the Raman scattering spectrum, which affects the damping constant. Therefore, high-entropy can be used to change the infrared emissivity through modulation of the damping constant.

## 4. Conclusions

In this study, LaB_6_ and two medium- and high-entropy hexaborides of (La_0.25_Sm_0.25_Ce_0.25_Eu_0.25_)B_6_ (MEREB_6_) and (La_0.2_Sm_0.2_Ce_0.2_Eu_0.2_Ba_0.2_)B_6_ (HEREB_6_) were designed and synthesized via a high-temperature solid-state reaction method with argon gas at 1600 °C. The XRD results provided evidence that the phases of these ME/HEREB_6_ ceramics were pure with no detectable impurity phase. XRD and EDS showed that ME/HEREB_6_ formed a homogeneous single phase solid solution. By analyzing the phase composition and microstructure, we showed that the ME/HEREB_6_ powder had a CsCl-type structure with Pm-3m space group with high crystallinity. The solar absorptivity of MEREB_6_ and HEREB_6_ is significantly increased in the range of 0.28–2.5 μm. The increase in solar absorptivity is mainly due to the decrease in free electron concentration and plasma frequency. Moreover, the emissivities of MEREB_6_ and HEREB_6_ in the ranges of 0.76–2.5 μm and 1–22 μm were also improved. The increase in emissivity was mainly caused by the multicomponent design, the induced lattice distortion, and the lower conductivity, resulting in an increase in the damping constant. In summary, both ME/HEREB_6_ ceramics had solar absorptivities greater than 80% and infrared emissivities close to 90%. Finally, in this study, ME/HEREB_6_ was synthesized, had high solar absorptivity and high infrared emissivity, and is expected to be used in solid particle solar receivers. Our study provides a new strategy for the development of solid particle solar receivers.

## Figures and Tables

**Table 1:** Calculated atomic size differences (*δ_r_*) and configuration entropy of medium- and high-entropy rare earth hexaborides.

Composition Code	*δ_r_* (%)	Δ*S_conf_*
MEREB_6_	2.58	1.3863 R
HEREB_6_	3.95	1.6094 R

**Table 2:** Constituent of raw materials of LaB_6_, MEREB_6_, and (HEREB_6_).

Composition Code	Molar Ratio of Raw Materials
LaB_6_	1La_2_O_3_:3B_4_C
MEREB_6_	1La_2_O_3_:1Sm_2_O_3_: 2CeO_2_:1Eu_2_O_3_:12B_4_C
HEREB_6_	1La_2_O_3_:1Sm_2_O_3_:2CeO_2_:1Eu_2_O_3_:2BaCO_3_:15B_4_C

**Table 3:** Lattice parameters and refined bond lengths (Å).

Samples	Lattice Parameters	Interatomic Distances (Å)		
	a (Å) = b (Å) = c (Å)	B–B_inter_ Mean	B-B_intra_ Mean	M-B Mean
LaB_6_	4.1576	1.647 (14)	1.775 (10)	3.0531 (18)
MEREB_6_	4.15546	1.672 (10)	1.756 (7)	3.0550 (13)
HEREB_6_	4.16005	1.677 (11)	1.756 (8)	3.0588 (15)

## Data Availability

Data are contained within the article.

## References

[1] Yao S., Wei Y., Lu Z., Guo S., Chen J., Yu Z. (2023). Review of Research Progress on Concentrated Solar Energy Utilization System. Renewables.

[2] Mehos M., Turchi C., Vidal J., Wagner M., Kruizenga A., Clifford H., William K., Charles A., Kruizenga A. (2017). Concentrating Solar Power Gen3 Demonstration Roadmap.

[3] He Y.-L., Qiu Y., Wang K., Yuan F., Wang W.-Q., Li M.-J., Guo J.-Q. (2020). Perspective of concentrating solar power. Energy.

[4] Ho C.K., Christian J.M., Yellowhair J., Armijo K., Nguyen C. Performance Evaluation of a High-Temperature Falling Particle Receiver. Proceedings of the ASME 2016 14th International Conference on Fuel Cell Science, Engineering and Technology.

[5] Le Gal A., Grange B., Casanova M., Perez A., Baltus W., Tessonneaud M., Flamant G. (2023). Experimental results for a MW-scale fluidized particle-in-tube solar receiver in its first test campaign. Sol. Energy.

[6] Nie F., Cui Z., Bai F., Wang Z. (2019). Properties of solid particles as heat transfer fluid in a gravity driven moving bed solar receiver. Sol. Energy Mater. Sol. Cells.

[7] Saeed R.S., Alswaiyd A., Saleh N.S., Alaqel S., Djajadiwinata E., El-Leathy A., Danish S.N., Al-Ansary H., Jeter S., Al-Suhaibani Z. (2022). Characterization of Low-Cost Particulates Used as Energy Storage and Heat-Transfer Medium in Concentrated Solar Power Systems. Materials.

[8] Chung K.M., Chen R. (2023). Black coating of quartz sand towards low-cost solar-absorbing and thermal energy storage material for concentrating solar power. Sol. Energy.

[9] Baumann T., Zunft S. (2015). Properties of granular materials as heat transfer and storage medium in CSP application. Sol. Energy Mater. Sol. Cells.

[10] Wu J., Zhang Z., Xu X., Ma S., Li P., Shi X. (2023). Preparation and Thermal Shock Resistance of Mullite Ceramics for High Temperature Solar Thermal Storage. J. Wuhan Univ. Technol.-Mater. Sci. Ed..

[11] Diago M., Iniesta A.C., Soum-Glaude A., Calvet N. (2018). Characterization of desert sand to be used as a high-temperature thermal energy storage medium in particle solar receiver technology. Appl. Energy.

[12] Díaz-Heras M., Calderón A., Navarro M., Almendros-Ibáñez J.A., Fernández A.I., Barreneche C. (2021). Characterization and testing of solid particles to be used in CSP plants: Aging and fluidization tests. Sol. Energy Mater. Sol. Cells.

[13] Radwan O.A., Humphrey J.D., Hakeem A.S., Zeama M. (2021). Evaluating properties of Arabian desert sands for use in solar thermal technologies. Sol. Energy Mater. Sol. Cells.

[14] Siegel N.P., Gross M.D., Coury R. (2015). The Development of Direct Absorption and Storage Media for Falling Particle Solar Central Receivers. J. Sol. Energy Eng..

[15] Zhao W., Sun Z., Alwahabi Z.T. (2020). Emissivity and absorption function measurements of Al_2_O_3_ and SiC particles at elevated temperature for the utilization in concentrated solar receivers. Sol. Energy.

[16] Kang Q., Flamant G., Dewil R., Baeyens J., Zhang H.L., Deng Y.M. (2019). Particles in a circulation loop for solar energy capture and storage. Particuology.

[17] Jiang K., Du X., Kong Y., Xu C., Ju X. (2019). A comprehensive review on solid particle receivers of concentrated solar power. Renew. Sustain. Energy Rev..

[18] Chao L., Bao L., Wei W., Tegus O. (2019). A review of recent advances in synthesis, characterization and NIR shielding property of nanocrystalline rare-earth hexaborides and tungsten bronzes. Sol. Energy.

[19] Bao L., Qi X., Tana T., Chao L., Tegus O. (2016). Synthesis, and magnetic and optical properties of nanocrystalline alkaline-earth hexaborides. CrystEngComm.

[20] Mattox T., Urban J. (2018). Tuning the Surface Plasmon Resonance of Lanthanum Hexaboride to Absorb Solar Heat: A Review. Materials.

[21] Zhang W., Zhao B., Ni N., Xiang H., Dai F.-Z., Wu S., Zhou Y. (2021). High entropy rare earth hexaborides/tetraborides (HEREB_6_/HE REB_4_) composite powders with enhanced electromagnetic wave absorption performance. J. Mater. Sci. Technol..

[22] Ji X.H., Zhang Q.Y., Xu J.Q., Zhao Y.M. (2011). Rare-earth hexaborides nanostructures: Recent advances in materials, characterization and investigations of physical properties. Prog. Solid State Chem..

[23] Sani E., Mercatelli L., Meucci M., Zoli L., Sciti D. (2017). Lanthanum hexaboride for solar energy applications. Sci. Rep..

[24] Zhang R.-Z., Reece M.J. (2019). Review of high entropy ceramics: Design, synthesis, structure and properties. J. Mater. Chem. A.

[25] Chen H., Xiang H., Dai F.-Z., Liu J., Zhou Y. (2019). Porous high entropy (Zr_0.2_Hf_0.2_Ti_0.2_Nb_0.2_Ta_0.2_)B_2_: A novel strategy towards making ultrahigh temperature ceramics thermal insulating. J. Mater. Sci. Technol..

[26] Gild J., Zhang Y., Harrington T., Jiang S., Hu T., Quinn M.C., Mellor W.M., Zhou N., Vecchio K., Luo J. (2016). High-Entropy Metal Diborides: A New Class of High-Entropy Materials and a New Type of Ultrahigh Temperature Ceramics. Sci. Rep..

[27] Chen H., Xiang H., Dai F.-Z., Liu J., Lei Y., Zhang J., Zhou Y. (2019). High porosity and low thermal conductivity high entropy (Zr_0.2_Hf_0.2_Ti_0.2_Nb_0.2_Ta_0.2_)C. J. Mater. Sci. Technol..

[28] Kovalev D.Y., Kochetov N.A., Chuev I.I. (2021). Fabrication of high-entropy carbide (TiZrHfTaNb)C by high-energy ball milling. Ceram. Int..

[29] He C.-Y., Zhao P., Gao X.-H., Liu G., La P.-Q. (2022). High-entropy alloy nitride nanofilms via a co-sputtering method enable superior optical performance and thermal robustness. Mater. Lett..

[30] He C.-Y., Gao X.-H., Yu D.-M., Zhao S.-S., Guo H.-X., Liu G. (2021). Toward high-temperature thermal tolerance in solar selective absorber coatings: Choosing high entropy ceramic HfNbTaTiZrN. J. Mater. Chem. A.

[31] McCormick C.R., Schaak R.E. (2021). Simultaneous Multication Exchange Pathway to High-Entropy Metal Sulfide Nanoparticles. J. Am. Chem. Soc..

[32] Guo H.-X., Wang W.-M., He C.-Y., Liu B.-H., Yu D.-M., Liu G., Gao X.-H. (2021). Entropy-Assisted High-Entropy Oxide with a Spinel Structure toward High-Temperature Infrared Radiation Materials. ACS Appl. Mater. Interfaces.

[33] Xue Y., Zhao X., An Y., Wang Y., Gao M., Zhou H., Chen J. (2022). High-entropy (La_0.2_Nd_0.2_Sm_0.2_Eu_0.2_Gd_0.2_)_2_Ce_2_O_7_: A potential thermal barrier material with improved thermo-physical properties. J. Adv. Ceram..

[34] Zhu H., Liu L., Xiang H., Dai F.-Z., Wang X., Ma Z., Liu Y., Zhou Y. (2022). Improved thermal stability and infrared emissivity of high-entropy REMgAl_11_O_19_ and LaMAl_11_O_19_ (RE=La, Nd, Gd, Sm, Pr, Dy; M=Mg, Fe, Co, Ni, Zn). J. Mater. Sci. Technol..

[35] Wang Q., Zhang Q., Wang G., Zhang Y., Xia M. (2022). High-entropy La(Fe_0.2_Co_0.2_Ni_0.2_Cr_0.2_Mn_0.2_)O_3_ ceramic exhibiting high emissivity and low thermal conductivity. Int. J. Appl. Ceram. Technol..

[36] Song J., Cheng Y., Xiang H., Dai F.-Z., Dong S., Chen G., Hu P., Zhang X., Han W., Zhou Y. (2023). Medium and high-entropy transition mental disilicides with improved infrared emissivity for thermal protection applications. J. Mater. Sci. Technol..

[37] Zhang W., Zhao B., Xiang H., Dai F.-Z., Wu S., Zhou Y. (2020). One-step synthesis and electromagnetic absorption properties of high entropy rare earth hexaborides (HEREB_6_) and high entropy rare earth hexaborides/borates (HEREB_6_/HEREBO_3_) composite powders. J. Adv. Ceram..

[38] Min G., Zheng S., Zou Z., Yu H., Han J. (2002). Reaction synthesis and formation mechanism of barium hexaboride. J. Ceram..

[39] Rodríguez-Carvajal J. (1993). Recent advances in magnetic structure determination by neutron powder diffraction. Phys. B Condens. Matter.

[40] Zhou Y., Dai F., Xiang H., Liu B., Feng Z. (2017). Shear anisotropy: Tuning high temperature metal hexaborides from soft to extremely hard. J. Mater. Sci. Technol..

[41] Bai L., Ma N., Liu F. (2009). Structure and chemical bond characteristics of LaB_6_. Phys. B Condens. Matter.

[42] Chao L., Bao L., Wei W., Tegus O., Zhang Z. (2015). Effects of Nanoparticle Shape and Size on Optical Properties of LaB_6_. Plasmonics.

[43] Zhou Y., Liu B., Xiang H., Feng Z., Li Z. (2015). YB_6_: A ‘Ductile’ and Soft Ceramic with Strong Heterogeneous Chemical Bonding for Ultrahigh-Temperature Applications. Mater. Res. Lett..

[44] Zhang Y., Wang L., Duan Y., Liu B., Liang J. (2022). Preparation and performance of Ce-doped far-infrared radiation ceramics by single iron ore tailings. Ceram. Int..

[45] Johnson R.W., Daane A.H. (1963). Electron Requirements of Bonds in Metal Borides. J. Chem. Phys..

[46] Chazalviel J.N., Campagna M., Wertheim G.K., Schmidt P.H. (1976). Study of valence mixing in SmB_6_ by X-ray photoelectron spectroscopy. Phys. Rev. B.

[47] Aita O., Watanabe T., Fujimoto Y., Tsutsum K. (1982). Sm M_4,5_ Spectra of Metallic Samarium and Samarium Hexaboride. J. Phys. Soc. Jpn..

[48] Pan X.-J., Bao L.-H., Ning J., Zhao F.-Q., Chao L.-M., Liu Z.-Z. (2021). Synthesis and optical absorption properties of nanocrystalline rare earth hexaborides Nd_1-x_Eu_x_B_6_ powders. Acta Phys. Sin..

[49] Luo X., Luo L., Zhao X., Cai H., Duan S., Xu C., Huang S., Jin H., Hou S. (2022). Single-phase rare-earth high-entropy zirconates with superior thermal and mechanical properties. J. Eur. Ceram. Soc..

[50] Toby B.H. (2012). R factors in Rietveld analysis: How good is good enough?. Powder Diffr..

[51] Lee O., Lee M., Choi Y., Sugio K., Matsugi K., Sasaki G. (2014). Microstructure Observation of Preform for High Performance VGCF/Aluminum Composites. Mater. Trans..

[52] Qiu J., Luo T., Yan Y., Xia F., Yao L., Tan X., Yang D., Tan G., Su X., Wu J. (2021). Enhancing the Thermoelectric and Mechanical Properties of Bi_0.5_Sb_1.5_Te_3_ Modulated by the Texture and Dense Dislocation Networks. ACS Appl. Energy Mater..

[53] Ye Y.F., Zhang Y.H., He Q.F., Zhuang Y., Wang S., Shi S.Q., Hu A., Fan J., Yang Y. (2018). Atomic-scale distorted lattice in chemically disordered equimolar complex alloys. Acta Mater..

[54] Dai F.-Z., Wen B., Sun Y., Xiang H., Zhou Y. (2020). Theoretical prediction on thermal and mechanical properties of high entropy (Zr_0.2_Hf_0.2_Ti_0.2_Nb_0.2_Ta_0.2_)C by deep learning potential. J. Mater. Sci. Technol..

[55] Aly K.A., Khalil N.M., Algamal Y., Saleem Q.M.A. (2016). Lattice strain estimation for CoAl_2_O_4_ nano particles using Williamson-Hall analysis. J. Alloys Compd..

[56] Liu X., Zhang P., Huang M., Han Y., Xu N., Li Y., Zhang Z., Pan W., Wan C. (2023). Effect of lattice distortion in high-entropy RE_2_Si_2_O_7_ and RE_2_SiO_5_ (RE=Ho, Er, Y, Yb, and Sc) on their thermal conductivity: Experimental and molecular dynamic simulation study. J. Eur. Ceram. Soc..

[57] Teredesai P., Muthu D.V.S., Chandrabhas N., Meenakshi S., Vijayakumar V., Modak P., Rao R.S., Godwal B.K., Sikka S.K., Sood A.K. (2004). High pressure phase transition in metallic LaB_6_: Raman and X-ray diffraction studies. Solid State Commun..

[58] Mukherjee A., Gulnar A.K., Sahoo D.K., Krishnamurthy N. (2012). Gas solid techniques for preparation of pure lanthanum hexaboride. Rare Met..

[59] Mattox T.M., Chockkalingam S., Roh I., Urban J.J. (2016). Evolution of Vibrational Properties in Lanthanum Hexaboride Nanocrystals. J. Phys. Chem. C.

[60] Yu Y., Wang S., Li W., Chen H., Chen Z. (2018). Synthesis of single-crystalline lanthanum hexaboride nanocubes by a low temperature molten salt method. Mater. Chem. Phys..

[61] Massidda S., Monnier R., Stoll E. (2000). Electronic structure of barium hexaboride. Eur. Phys. J. B.

[62] Singh N., Saini S.M., Nautiyal T., Auluck S. (2007). Electronic structure and optical properties of rare earth hexaborides RB_6_(R = La, Ce, Pr, Nd, Sm, Eu, Gd). J. Phys. Condens. Matter.

[63] Kimura S., Nanba T., Kunii S., Suzuki T., Kasuya T. (1990). Anomalous infrared absorption in rare-earth hexaborides. Solid State Commun..

[64] (2022). Solar Energy—Reference Solar Spectral Irradiance at the Ground at Different Receiving Conditions—Part 1: Direct Normal and Hemispherical Solar Irradiance for Air Mass 1,5.

[65] Dong S., Zhang F., Li N., Zeng J., Liang P., Zhang H., Liao H., Jiang J., Deng L., Cao X. (2020). Thermal radiation and cycling properties of (Ca, Fe) or (Sr, Mn) co-doped La_2_Ce_2_O_7_ coatings. J. Eur. Ceram. Soc..

[66] Gerald D., Mahan K.R.S. (1990). Local Density Theory of Polarizability.

